# Advances in Autophagy, Tissue Injury, and Homeostasis: *Cells* Special Issue

**DOI:** 10.3390/cells8070743

**Published:** 2019-07-19

**Authors:** Pei-Hui Lin

**Affiliations:** 1 Davis Heart and Lung Research Institute, The Ohio State University, Columbus, OH 43210, USA; Pei-Hui.Lin@osumc.edu; 2 Department of Surgery, The Ohio State University Wexner Medical Center, Columbus, OH 43210, USA

Macroautophagy (hereafter referred to as autophagy, a word derived from Greek meaning “auto-digestion”) is a lysosome-dependent quality control process to degrade and turnover damaged or senescent organelles and proteins for cellular renewal. This essential process occurs in many eukaryotes to determine the cellular fitness and tissue homeostasis of organisms. Basal autophagy plays important roles during development and differentiation. Remarkably, autophagy is also a defense mechanism employed against environmental stress such as nutrient deprivation, aging, pathogen invasion, and various disease states [1]. As such, autophagy is an inducible and highly regulated process via a versatile regulatory network to intimately control several vital cellular responses, including inflammation, cell death, energy metabolism, organelles’ (mitochondria and others) homeostasis, and aging. Although the role of autophagy in the maintenance of tissue homeostasis is relatively better documented, its role during tissue injury and regeneration is still emerging. 

In this Special Issue, we focus on the roles of autophagy in systemic, specific tissue (organs and cells) injury or organ failure associated with sepsis, inflammation, metabolic disorder, toxic chemicals, ischemic–reperfusion, hypoxic oxidative stress, tissue fibrosis, trauma, nutrient starvation, cancer biology, and aging. This Special Issue contains 5 research papers and 10 review articles addressing the impact of autophagy on various organ injuries and homeostasis. Each of the reviews is authored by experts in their fields and our intention is to provide comprehensive updates in specific areas relating autophagy to tissue injury and homeostasis in which there has been considerable recent progress. The knowledge gained from the identification and characterization of new molecular mechanisms will shed light on biomedical applications for tissue protection through the modulation of autophagy.

Three articles focus on the role of mitochondrial ubiquitin kinase PINK1 and Parkin E3 ubiquitin ligase (PINK1/Parkin)-dependent mitophagy in organ homeostasis. Work by Zhou et al. [2] demonstrated the role of Notoginsenoside R1 (NGR1), a plant saponin extract, in ameliorating diabetic retinopathy through the PINK1-dependent activation of mitophagy and inhibition of apoptosis, oxidative stress in high glucose-stressed cultured rat retinal Müller cells (rMC-1) and retina tissue of *db/db* mice. Eid et al.’s [3] pioneering study elucidated the involvement of the PINK1/Parkin-dependent mitophagy pathway in acute ethanol intake-induced mitochondrial damage in Sertoli cells (SCs), the somatic cells of the testis which are essential for testis formation and spermatogenesis, in adult rats. This study is useful for the scientific community as it could help to define new therapeutic strategies by stimulating Parkin-mediated mitophagy in alcohol-related organ damage. Caloric restriction (or diet restriction, DR) is the best known strategy to robustly improve health, lifespan, and age-associated disease [4]. Diet restriction offers benefits against acute kidney injury (AKI) in young rats; however, such DR benefits are lost in aged animals encountering AKI due to the deterioration in the autophagy/mitophagy flux [5].

Metformin, a biguanide drug, is the most commonly prescribed drug for the treatment of type 2 diabetes as a glucose-lowering and insulin-sensitizing agent. Previous work has shown that metformin disrupts mitochondria energetics and represses the mechanistic target of rapamycin complex 1 (mTORC1) signaling in cancer cells [6]. Saladini et al. [7] demonstrated that the anti-tumoral action of metformin is due to the inhibition of glutaminase and autophagy has the potential to improve the efficacy of chemotherapy. Exosomes (and the containing paracrine factors) derived from mesenchymal stem/stromal cells (MSCs) have been demonstrated to hold great potential in regenerative medicine [8]. Ebrahim et al. [9] examined how MSC-derived exosomes attenuated diabetic nephropathy in a rat model of streptozotocin-induced diabetes through a mechanism of enhanced autophagy.

In the review articles, we included topics summarizing the current progress on the cardioprotective effects of autophagy in sepsis [10]. The specific activation of autophagy initiation factor Beclin-1 in protecting cardiac mitochondria, attenuating inflammation, and improving cardiac function in septic injury was discussed [10] (also see the comments in Reference [11]). Autophagy in various lung diseases, including acute lung injury (ALI), infectious disease, chronic obstructive pulmonary disease (COPD), idiopathic pulmonary fibrosis (IPF), pulmonary arterial hypertension (PAH), cystic fibrosis (CF), and tuberculosis are discussed [12]. Lin et al. [13] discussed the current concepts of autophagy and its molecular pathophysiologies in different kidney cell types with AKI, chronic kidney disease, drug nephrotoxicity, and aging kidneys. Some therapeutics targeting autophagy in kidney diseases are also summarized. 

Two articles summarized the contribution of autophagy in the homeostasis and pathogenesis of the intestine, focusing on inflammatory bowel disease (IBD) from the aspects of intestinal innate immune cells response [14] and the clinical relevance of several autophagy-related genes (ATGs) in the pathogenesis of IBD [15]. These underscore the connection of autophagy in regulating innate immune functions such as inflammatory cytokines production and the cross-talk between various immune cells and intestine cells.

Weiskirchen and Tacke [16] excellently summarized the current knowledge on the role and mechanisms of autophagy in multiple liver cell types in health and disease. The normal hepatic functions such as gluconeogenesis, glycogenolysis, fatty acid oxidation, and disorders such as hereditary liver diseases, alcoholic liver disease, non-alcoholic fatty liver disease, hepatic fibrosis, and hepatocellular carcinoma (HCC) are discussed. Importantly, the opposing functions of autophagy in stage-specific pathogenesis in fibrosis and HCC are also discussed. The dual roles of autophagy in HCC is further supported by Yazdani et al. [17]. Both pro- and anti-tumorigenic autophagy are described for HCC. Therefore, it is critical to concisely develop autophagy-related pharmacological target therapies.

Lee et al. [18] offer a timely summarization of autophagy in skeletal muscle regeneration in aging. As the skeletal muscle is the largest organ in the body with remarkable regenerative capacity and regulation of energy metabolism and body activities, autophagy critically impacts muscle physiology. The effects of aging on autophagy, the role of myofibers, satellite (stem) cells as well as the immune system (mainly macrophages) during muscle repair/regeneration are discussed. Some rejuvenation strategies that alter autophagy to improve muscle regenerative function are also proposed. Sanchez et al. [19] reviewed the current knowledge on physical exercise’s role in the regulation of cellular component turnover through multiple mechanisms involving autophagy, organelles’ quality control, energy sensors, and anabolic signaling. This knowledge is critical in the design of exercise regiments and nutritional interventions and the development of countermeasures during illness.

Finally, Wu et al. [20] discussed the recent development of dual roles, both beneficial and detrimental, of autophagy to neurotrauma after spinal cord and brain injury (SCI/TBI). It is suggested that impairment of autophagic flux could serve as a secondary injury process of SCI/TBI. Moreover, modulation of the autophagy–lysosomal pathway could be with therapeutic potential in neurotrauma and neuroinflammation conditions.

The 15 publications in this Special Issue summarize the significant amount of progress that has contributed to our understanding of autophagy in normal tissue homeostasis and in disease states during dysfunction. Importantly, these publications provide future research directions for the design of therapeutic strategies targeting autophagy to combat disease and tissue injuries. I wish to thank all the authors for their contributions, the scientific communities for peer reviewing, and the staff at the *Cells* editorial office for their work on this Special Issue.

## References

[1] Giampieri F., Afrin S., Forbes-Hernandez T.Y., Gasparrini M., Cianciosi D., Reboredo-Rodriguez P., Varela-Lopez A., Quiles J.L., Battino M. (2019). Autophagy in Human Health and Disease: Novel Therapeutic Opportunities. Antioxid. Redox Signal..

[2] Zhou P., Xie W., Meng X., Zhai Y., Dong X., Zhang X., Sun G., Sun X. (2019). Notoginsenoside R1 Ameliorates Diabetic Retinopathy through PINK1-Dependent Activation of Mitophagy. Cells.

[3] Eid N., Ito Y., Horibe A., Otsuki Y., Kondo Y. (2019). Ethanol-Induced Mitochondrial Damage in Sertoli Cells is Associated with Parkin Overexpression and Activation of Mitophagy. Cells.

[4] Madeo F., Carmona-Gutierrez D., Hofer S.J., Kroemer G. (2019). Caloric Restriction Mimetics against Age-Associated Disease: Targets, Mechanisms, and Therapeutic Potential. Cell Metab..

[5] Andrianova N.V., Jankauskas S.S., Zorova L.D., Pevzner I.B., Popkov V.A., Silachev D.N., Plotnikov E.Y., Zorov D.B. (2018). Mechanisms of Age-Dependent Loss of Dietary Restriction Protective Effects in Acute Kidney Injury. Cells.

[6] Wu L., Zhou B., Oshiro-Rapley N., Li M., Paulo J.A., Webster C.M., Mou F., Kacergis M.C., Talkowski M.E., Carr C.E. (2016). An Ancient, Unified Mechanism for Metformin Growth Inhibition in C. elegans and Cancer. Cell.

[7] Saladini S., Aventaggiato M., Barreca F., Morgante E., Sansone L., Russo M.A., Tafani M. (2019). Metformin Impairs Glutamine Metabolism and Autophagy in Tumour Cells. Cells.

[8] Zhao T., Sun F., Liu J., Ding T., She J., Mao F., Xu W., Qian H., Yan Y. (2019). Emerging Role of Mesenchymal Stem Cell-derived Exosomes in Regenerative Medicine. Curr. Stem Cell Res. Ther..

[9] Ebrahim N., Ahmed I.A., Hussien N.I., Dessouky A.A., Farid A.S., Elshazly A.M., Mostafa O., Gazzar W.B.E., Sorour S.M., Seleem Y. (2018). Mesenchymal Stem Cell-Derived Exosomes Ameliorated Diabetic Nephropathy by Autophagy Induction through the mTOR Signaling Pathway. Cells.

[10] Sun Y., Cai Y., Zang Q.S. (2019). Cardiac Autophagy in Sepsis. Cells.

[11] Abdellatif M., Sedej S., Madeo F., Kroemer G. (2018). Cardioprotective effects of autophagy induction in sepsis. Ann. Transl. Med..

[12] Wang K., Chen Y., Zhang P., Lin P., Xie N., Wu M. (2019). Protective Features of Autophagy in Pulmonary Infection and Inflammatory Diseases. Cells.

[13] Lin T.A., Wu V.C., Wang C.Y. (2019). Autophagy in Chronic Kidney Diseases. Cells.

[14] Iida T., Yokoyama Y., Wagatsuma K., Hirayama D., Nakase H. (2018). Impact of Autophagy of Innate Immune Cells on Inflammatory Bowel Disease. Cells.

[15] Kim S., Eun H.S., Jo E.K. (2019). Roles of Autophagy-Related Genes in the Pathogenesis of Inflammatory Bowel Disease. Cells.

[16] Weiskirchen R., Tacke F. (2019). Relevance of Autophagy in Parenchymal and Non-Parenchymal Liver Cells for Health and Disease. Cells.

[17] Yazdani H.O., Huang H., Tsung A. (2019). Autophagy: Dual Response in the Development of Hepatocellular Carcinoma. Cells.

[18] Lee D.E., Bareja A., Bartlett D.B., White J.P. (2019). Autophagy as a Therapeutic Target to Enhance Aged Muscle Regeneration. Cells.

[19] Sanchez A.M., Candau R., Bernardi H. (2019). Recent Data on Cellular Component Turnover: Focus on Adaptations to Physical Exercise. Cells.

[20] Wu J., Lipinski M.M. (2019). Autophagy in Neurotrauma: Good, Bad, or Dysregulated. Cells.

